# Quantifying the relationship between climatic indicators and leptospirosis incidence in Fiji: A modelling study

**DOI:** 10.1371/journal.pgph.0002400

**Published:** 2023-10-11

**Authors:** Eleanor M. Rees, Martín Lotto Batista, Mike Kama, Adam J. Kucharski, Colleen L. Lau, Rachel Lowe

**Affiliations:** 1 Centre for Mathematical Modelling of Infectious Diseases, London School of Hygiene and Tropical Medicine, London, United Kingdom; 2 Centre on Climate Change and Planetary Health, London School of Hygiene and Tropical Medicine, London, United Kingdom; 3 Barcelona Supercomputing Center (BSC), Barcelona, Spain; 4 Epidemiology Department, Helmholtz Centre for Infection Research, Brunswick, Germany; 5 Fiji Centre for Communicable Disease Control, The University of the South Pacific, Suva, Fiji; 6 School of Public Health, Faculty of Medicine, The University of Queensland, Herston, Queensland, Australia; 7 Catalan Institution for Research and Advanced Studies (ICREA), Barcelona, Spain; Yale University School of Medicine, UNITED STATES

## Abstract

Leptospirosis, a global zoonotic disease, is prevalent in tropical and subtropical regions, including Fiji where it’s endemic with year-round cases and sporadic outbreaks coinciding with heavy rainfall. However, the relationship between climate and leptospirosis has not yet been well characterised in the South Pacific. In this study, we quantify the effects of different climatic indicators on leptospirosis incidence in Fiji, using a time series of weekly case data between 2006 and 2017. We used a Bayesian hierarchical mixed-model framework to explore the impact of different precipitation, temperature, and El Niño Southern Oscillation (ENSO) indicators on leptospirosis cases over a 12-year period. We found that total precipitation from the previous six weeks (lagged by one week) was the best precipitation indicator, with increased total precipitation leading to increased leptospirosis incidence (0.24 [95% CrI 0.15–0.33]). Negative values of the Niño 3.4 index (indicative of La Niña conditions) lagged by four weeks were associated with increased leptospirosis risk (-0.2 [95% CrI -0.29 –-0.11]). Finally, minimum temperature (lagged by one week) when included with the other variables was positively associated with leptospirosis risk (0.15 [95% CrI 0.01–0.30]). We found that the final model was better able to capture the outbreak peaks compared with the baseline model (which included seasonal and inter-annual random effects), particularly in the Western and Northern division, with climate indicators improving predictions 58.1% of the time. This study identified key climatic factors influencing leptospirosis risk in Fiji. Combining these results with demographic and spatial factors can support a precision public health framework allowing for more effective public health preparedness and response which targets interventions to the right population, place, and time. This study further highlights the need for enhanced surveillance data and is a necessary first step towards the development of a climate-based early warning system.

## Introduction

Leptospirosis is a zoonotic disease with an estimated 1.03 million cases, and 58,000 deaths reported globally each year [1]. It is found in all regions of the world, but the burden of disease is particularly high in Oceania and other resource-limited settings [2–4], and it is a major public health concern in many countries. It is caused by pathogenic spirochaete bacteria, of the genus *leptospira* [5]. Leptospirosis typically presents as an acute febrile illness and symptoms can resemble other diseases, such as dengue and malaria, which often leads to misdiagnosis or underdiagnosis [5–9]. In some patients, more severe disease can occur and the case fatality rate of leptospirosis is estimated to be approximately 7% [1], although in settings with limited access to treatment and diagnosis it can be higher. Surveillance of leptospirosis is often limited, and many countries have limited capacity for diagnostic testing. Furthermore, the laboratory diagnosis of leptospirosis is challenging, and there is a lack of adequate diagnostic tests available for accurate and early diagnosis of leptospirosis [10,11].

Leptospirosis transmission is driven by complex interactions between animals, humans and their environment. Hundreds of animal species have been identified as hosts for leptospirosis, including rodents, livestock, and domestic and wild animals [7,12,13]. Humans can become infected, either directly through contact with infected animals or tissue, or indirectly through water or soil contaminated by animal urine; but human-to-human transmission is extremely rare [5,7,8,14]. Once in the environment, bacteria can survive for weeks or even months in water or moist soil [5,15–17]. As such, there are many different risk factors for leptospirosis, and these are context specific. Risk factors include occupational (such as agricultural workers and abattoir workers), lack of sanitation, poor living conditions, animals in the community and recreational exposures [4,18–20].

Climatic factors have also been shown to increase leptospirosis risk, with outbreaks of leptospirosis often associated with extreme precipitation and flooding events [4,19,20]. Extreme precipitation and flooding bring humans into increased contact with the bacteria and their animal hosts, as well as disrupting public health infrastructure and sanitation networks. Furthermore, *Leptospira* can survive for longer periods in water and moist soil, and flooding prevents animal urine from being absorbed into the soil or evaporation. These outbreaks have been reported worldwide in geographically diverse areas, although they appear to be more common in tropical island nations and resource-poor settings [4,20]. Temperature may also have a role in leptospirosis transmission since higher temperatures and humid environments have been shown to prolong *Leptospira* environmental survival [5,15,16]. Human interaction with the environment also increases with higher temperatures, leading to more exposure risk [4]. However, there is evidence that this relationship may not be linear, as very high temperatures (>42°C) have been shown to reduce *Leptospira* survival [21,22].

Large-scale climate patterns, such as the El Niño-Southern Oscillation (ENSO), which is an inter-annual cycle involving changes in sea-surface temperature (SST) in the central and eastern tropical Pacific Ocean, influences the timing and intensity of rainfall in the tropical Pacific islands and elsewhere. El Niño and La Niña are opposite phases of the ENSO, and on average ENSO events occur every four years. In Fiji, El Niño tends to be associated with drier and cooler conditions than normal and can be associated with droughts in some parts of the country, whilst La Niña is associated with wetter than normal conditions. This leads to increased incidence of flooding, particularly if the La Niña event coincides with the wet season [23]. However, since Fiji lies in the transition zone, the impacts of ENSO are not always uniform and no two ENSO events are quite the same, although they tend to share typical characteristics. ENSO has been shown to be associated with leptospirosis outbreaks in New Caledonia [24]. The authors found that La Niña phases (cool SST anomalies in the Pacific Ocean) were associated with leptospirosis outbreaks, and they demonstrated how Sea Surface Temperature (SST) anomalies (a measure of ENSO) may be used as an early warning system in this setting. Tropical cyclones regularly occur in Fiji, usually during the wet season (November to April), and these can cause extensive damage and flooding [23,25]. On average, 1–2 cyclones affect Fiji every season, and tropical cyclone activity has been shown to increase during El Niño phases, compensated by a decrease during La Niña phases [23,25]. Due to climate change, Fiji is expected to experience more extreme rainfall events and rising temperatures. Furthermore, tropical cyclones are expected to be less frequent, but more intense in the future [26]. Since the effects of ENSO vary globally, the relationship between leptospirosis incidence and ENSO is likely to vary depending on setting. A study in New Caledonia found that La Niña phases (cool SST anomalies in the Pacific Ocean) were associated with leptospirosis outbreaks [24]. By contrast, another study in Argentina showed the El Nino phase (warm SST anomalies in the Pacific Ocean) were associated with leptospirosis incidence, and they demonstrated how the Nino 3.4 index could form part of an early warning system in this setting [27].

Leptospirosis disease burden is particularly high in Oceania, and a systematic review found that Oceania had the largest per capita leptospirosis morbidity (150.68 cases per 100,000 per year) and mortality (9.61 deaths per 100,000 per year) globally [2]. Leptospirosis is endemic in Fiji, with cases reported throughout the year. However, outbreaks of leptospirosis are reported most frequently during the rainy season (between January and March) [28]. In recent years the number of reported cases has been increasing, with large outbreaks occurring more frequently. This could either be due to a real increase in the number of cases, or improvements in testing capabilities and increased health awareness, particularly since the release of new leptospirosis guidelines in 2016. A previous seroprevalence study conducted in 2013 identified risk factors associated with leptospirosis cases in Fiji, including individual risk factors (working outdoors, male sex and iTaukei ethnicity) and community risk factors (lack of treated water at home, living in rural areas, high poverty rate, living less than 100m from a major river, pigs in the community and high cattle density) [18]. The seroprevalence study was further analysed and it was found that there was significant geographical variation in these sociodemographic and environmental drivers [29].

Despite the substantial disease burden, there still is limited quantitative evidence about the effects of precipitation, temperature and ENSO on leptospirosis cases in the Pacific region. This study aims to identify the most relevant climatic indicators, including different precipitation indicators (e.g. total precipitation), temperature and the Niño 3.4 index (based on SST in the Pacific Ocean), and quantify the effect of these indicators at different spatial and temporal scales on leptospirosis incidence in Fiji. Together with the knowledge of demographic and spatial factors, this can support a precision public health approach, efficiently targeting interventions to the right populations at the most appropriate time and place. This will become increasingly important in the future as extreme weather events are expected to increase in frequency as a result of climate change, the effects of which are already being felt in this region.

## Methods

### Ethics statement

This study is a secondary analysis of data collected as part of routine surveillance of leptospirosis in Fiji. The research was approved by the London School of Hygiene and Tropical Medicine Research Ethics Committee (reference number 16171) and by the Fiji National Health Research and Ethics Review Committee (reference number 2019.72.NW).

### Study location and setting

**Fig 1:**
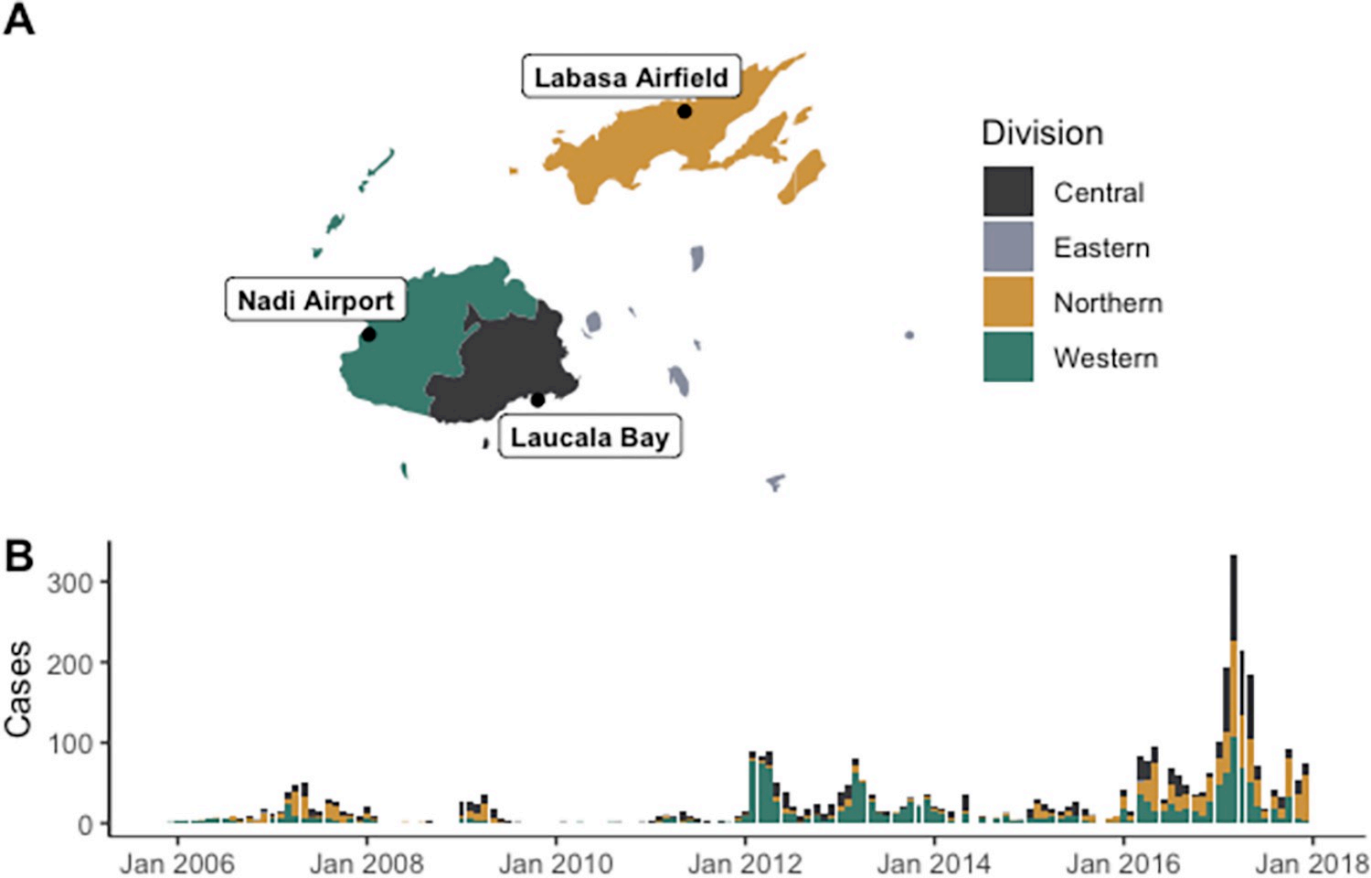
A. Map of divisions within Fiji. The location of the three meteorological stations used are labelled (Laucala Bay, Nadi Airport and Labasa Airfield). B. Monthly reported leptospirosis cases by division between 2006 and 2017 in Fiji. Map created with Natural Earth (https://www.naturalearthdata.com/).

Fiji is an island nation in the South Pacific Ocean and is made up of over 330 islands. It is classified by the United Nations as a small island developing state [30] with a per capita GDP of US$6,143 in 2019 [31] (Fig 1A). The population size was 884 887 in 2017 [32], and it is estimated that 90% of the population in Fiji are coastal dwellers [33]. Fiji is divided into four administrative divisions (Central, Western, Northern and Eastern), which have population sizes of 378 284, 337 041, 131 914 and 37 648, respectively (32). There are two main islands in Fiji; Viti Levu is split between the Central and Western division and approximately 80% of the population resides on this island, and Vanua Levu in the Northern division. The capital, Suva, is located in the South East of the Central division, and has a population size of 94 088. The percentage of the population living in urban areas has been increasing over time, with 55.9% urban dwellers in 2017, an increase of 16.3% since 2007 [32]. The percentage of the population living in urban areas is highest in the Central division (71.3%), followed by the Western division (53.8%), Northern division (29.4%) and Eastern division (11.3%) [32]. The percentage of people living in informal housing in urban areas is highest in the Central (17.0%) and Western (15.0%) divisions, compared with the Northern division (4.3%) [32].

### Leptospirosis surveillance data

Leptospirosis is a notifiable disease in Fiji, and cases are reported through the Notifiable Diseases Surveillance System. Cases are defined as suspected, probable, and confirmed. Suspected cases are based entirely on clinical assessment and epidemiological risk factors. Probable and confirmed cases are based on a combination of clinical assessment, epidemiological risk factors and the results from diagnostic testing. An outbreak of leptospirosis is defined in Fiji when the incidence rate is >2 standard deviations above the average of the last five non-outbreak years [34]. The two most common diagnostic tests performed in Fiji are the anti-Leptospira immunoglobulin M (IgM) enzyme-linked immunosorbent assay (ELISA), which is only available from Mataika house in Suva, and rapid diagnostic tests, which are available in laboratories across Fiji. Cases which are positive using these tests are considered probable cases. Very few cases in Fiji are confirmed using confirmatory diagnostic tests. Further information can be found in the clinical guidelines [35]. Once an outbreak has been declared, it recommended that diagnosis is performed based on case definitions and clinical assessment to conserve testing capabilities [34].

In this analysis, ELISA-positive cases of leptospirosis reported between 2006 and 2017 were included. Where possible, date of sample collection was used. For 225 cases, the date of sample collection was unavailable, and the date received or date tested were used instead. These dates were then adjusted by the median number of days between date of sample collection and date received (four days) and the date of sample collection and date tested (16 days). Cases were aggregated into weekly (by ISO weeks) and monthly cases.

Fiji experiences coinciding outbreaks of leptospirosis and dengue, which share clinical manifestations. To explore the possible impact of dengue on leptospirosis surveillance, we looked at prolonged fever from the World Health Organization (WHO) Pacific Syndromic Surveillance System [36] between 2013 to 2017 (which is any fever lasting three or more days, which could be an indication of leptospirosis or dengue fever; Fig A in S1 Text). We find that the syndromic surveillance generally aligns with the upticks in reported leptospirosis cases. The two main discrepancies in the time series occur in early 2014, where the large peak in prolonged fever–but not leptospirosis cases–coincided with a large dengue-3 epidemic, and in mid-2017, which coincided with a smaller dengue-2 outbreak. This suggests that the leptospirosis case time series is not clearly influenced by occasional overlap with dengue epidemics, unlike the prolonged fever data.

### Demographic data

Population estimates for each division were obtained from the Fiji Bureau of Statistics from the 2007 and 2017 Population and Housing Census [32]. Linear interpolation was used to estimate the population size between these two time periods for each division.

### Meteorological data

The analysis was performed at both the weekly and monthly time scale, therefore daily climatic data was aggregated to both time scales. Daily precipitation, minimum daily temperature and maximum daily temperature were obtained from the Fiji meteorological services. Exploratory analysis was performed to select one meteorological station in each division, based on data completeness for the study period. These were Laucala Bay (Central division; latitude: -18.13, longitude: 178.45), Nadi Airport (Western division; latitude: -17.75, longitude: 177.43) and Labasa Airfield (Northern division; latitude: -16.47, longitude: 179.33; Fig 1B). We also explored using averages over all meteorological stations in a division and found that there was little impact on the results obtained, therefore for simplicity we selected one station per division. Weekly and monthly Optimum Interpolation Sea Surface temperature (SST) version 2.1 (OISSTV2.1) anomalies in region 3.4 (Niño 3.4 index) and region 4 (Niño 4 index) were obtained from the National Oceanic and Atmospheric Administration (NOAA) [37].

To capture extreme precipitation, five precipitation indicators were chosen based on descriptive indices defined by the World Meteorological Organisation Expert Team on Climate Change Detection and Indices [38]. These five indicators are described in Table 1. Different durations of the time period (*j*) were tested, (two, four, six and eight weeks for the weekly surveillance data, and one, two and three months for the monthly surveillance data).

**Table 1:** Definition of precipitation indicators defined and adapted from the Expert Team on Climate Change Detection and Indices [38].

Precipitation indicator	Definition
Total precipitation (TP)	Total precipitation (TP) on wet days (≥ 1mm). Let P_wj_ be the daily precipitation amount on a wet day *w* (P ≥ 1 mm) in period *j*. Then, *Total precipitation*_*j*_ *= sum (P*_*wj*_*)*
Heavy precipitation days (P10)	Count of days where daily precipitation amount (P) ≥ 10 mm Let P_ij_ be the daily precipitation amount on day *i* in period *j*. Count the number of days where P_ij_ ≥ 10 mm.
Very heavy precipitation days (P20)	Count of days where daily precipitation amount (P) ≥ 20 mm Let P_ij_ be the daily precipitation amount on day *i* in period *j*. Count the number of days where P_ij_ ≥ 20 mm.
Number of wet days (WD)	Count of days where daily precipitation amount (P) ≥ 1 mm Let P_ij_ be the daily precipitation amount on day *i* in period *j*. Count the number of days where P_ij_ ≥ 1 mm.
Mean consecutive wet days (CWD)	Mean number of CWD (consecutive wet days) (≥1mm) in period *j*. Let P_ij_ be the daily precipitation amount on day *i* in period *j*. Count the largest number of consecutive days where P_ij_ ≥ 1 mm. Then, *Mean consecutive wet days*_*j*_ *= mean (CWD*_*j*_*)*

Long-term periods of abnormal wetness can also be captured using the Standardised Precipitation Index (SPI) and the Standardised Precipitation Evapotranspiration Index (SPEI) [39,40]. Positive values of SPI and SPEI correspond to wet periods, whilst negative values correspond to dry periods. SPI and SPEI were calculated using the SPEI package in R [41]. SPI was calculated for each division using daily precipitation from 1990 to 2018 from three meteorological stations in Fiji (Laucala Bay, Nadi Airport and Labasa Airfield). To calculate SPEI, first minimum and maximum daily temperature, as well as latitude coordinates of the meteorological stations, were used to calculate monthly reference evapotranspiration (ETO) according to Hargreaves equation. Then the climatic water balance (d_i,j_), which provides a measure of the water surplus or deficit for a specific month ‘i’ in the year ‘j’, was calculated as precipitation minus reference evapotranspiration:

$$d_{i,j}=P_{i,j}-ETO_{i,j}$$

SPI and SPEI were calculated for different time scales, one, three and six months.

To account for the time between infection to symptom onset, as well as the delayed effects of climate indicators on disease, different time lags for temperature, precipitation indicators, SPI and SPEI were tested, from 1–12 weeks (weekly surveillance data) and 1–3 months (monthly surveillance data). Changes in SST anomalies can take longer to impact the local climate; therefore, longer time lags were used to assess the effect of Niño 3.4 and Niño 4 indices on leptospirosis transmission, from 1–20 weeks (and 1–4 months).

### Meteorological events

In Fiji, between 2007 and 2017 (the study period) there were six tropical cyclones and five major flooding events (with an additional five flooding events triggered by the tropical cyclones) recorded by the Emergency Events Database (EM-DAT [42]; Table A in S1 Text). Data on disasters were obtained from the EM-DAT database, which includes events if either there have been 10 or more deaths; more than 100 people affected, injured or homeless; or a declaration by the country of a state of emergency and/or an appeal for international assistance. The primary disaster may trigger another event (i.e., a tropical cyclone may trigger a flooding event), and this is recorded in Table A in S1 Text. The timing of the tropical cyclones and flooding events is displayed in Fig B in S1 Text, along with weekly precipitation data.

### Statistical analysis

First, we formulated a hierarchical mixed-effects model using counts of leptospirosis cases per week over 12 years (January 2006 to December 2017) in Fiji. Counts of leptospirosis cases, y_st_, (where *s* is division and *t* is time), were assumed to follow a negative binomial distribution to account for the overdispersion within the data, with mean μ and overdispersion parameter κ,

$$y_{st}|\mu_{st}∼NegBin(\mu_{st},\kappa )$$

which we modelled using the linear predictor,

$$log(\mu_{st})=log(P_{st})+\alpha +\Sigma \beta_{i}x_{ist}+\delta_{sa(t)}+\gamma_{w(t)}$$

where α is the model intercept and log(*P*_*st*_) is the population size per 100,000 per year per division, which we inputted as an offset. *Σβ*_*i*_*x*_*ist*_ is a vector of covariate climatic coefficients. To account for the seasonality of leptospirosis cases, a weekly random effect, *γ*_*w*(*t*)_, where *w(t)* = 1,…,52. This was modelled using a first-order random walk, which allows leptospirosis incidence rates in one week to depend on the previous week. Independent random effects for each year (*δ*_*s a*(*t*)_), 2006–2017 replicated by division were included to allow for additional sources of variation due to unobserved confounding factors such as variations in healthcare access, case reporting and changes in diagnostic capacity over time and between divisions.

Model parameters were estimated using Integrated Nested Laplace Approximation (INLA).

Model selection was performed using the widely applicable information criterion (WAIC), which balances model fit with model complexity, and therefore aims to balance the risks of overfitting and underfitting. Models with a lower WAIC indicate a more parsimonious model [43]. The cross-validated logarithmic score (CV log score) was also used to assess model fit. This is based on the conditional predictive ordinate (CPO) leave-one-out cross-validation score, where smaller values indicate greater predictive power of the model [44]. An R^2^_LR_ statistic was also calculated based on a likelihood ratio test of the deviance between the candidate model and the baseline model (seasonal and interannual random effects). R^2^_LR_ is useful as a measure of goodness-of-fit and provides an intuitive measure of the ability of the model to account for the variation in the dependent variable.

A baseline model was first developed, which included weekly and yearly random effects. Exploratory analysis and selection criteria were used to select the most appropriate time lags for the climate covariates, and a subset of covariates were chosen for further analysis. We then explored combining the different precipitation indicators with different temperature and ENSO measures. The final model was selected using the model selection criteria described above and comparing models of increasing complexity (with regard to input variables and model structure) to the baseline model. We were also interested in the differences between the divisions; therefore, the final model was fitted separately for each division.

To check for correlation and collinearity between variables, we calculated Pearson’s rank correlation index using the “corrplot” package in R [45]. A Pearson’s correlation r > 0.6 was considered to be high correlation. We also calculate the variance inflation factor (VIF) using the “car” package in R [46]. A VIF > 5 was considered to be indicative of high variance inflation.

Given the potential for heterogeneity in weekly data, we performed a sensitivity analysis to test how the model results changed at different spatial and temporal scales. We repeated the model formulation and selection, using counts of leptospirosis cases per month. Instead of using a weekly random effect, a monthly random effect was used, again using first order random walk. Independent random effects for each year (*δ*_*a*(*t*)*s*_), 2006–2017 replicated by division were included as before. Finally, we repeated the analysis for the whole country, instead of by division. As before we repeated the model formulation and selection, using counts of leptospirosis cases per month. Again, a monthly random effect was used, using first order random walk. This time, independent random effects for each year (*δ*_*t*_), 2006–2017 were included, not replicated by division.

We performed all analysis using R programming language version 4.1.1. Code and data are available on GitHub (https://github.com/erees/leptospirosis_Fiji_2023).

## Results

### Leptospirosis incidence in Fiji

Between 2006 and 2017, a total of 3,485 ELISA-positive cases of leptospirosis were reported in Fiji (979 in the Central division, 1,481 cases in the Western division, 1,019 cases in the Northern division and six in the Eastern division; Fig 1A and 1B). Since only six cases were reported in the Eastern division over the study period, the Eastern division was excluded from the analysis. Over this time period, the Northern division reported the highest case rates (759.9 cases per 100,000) followed by the Western division (452.0 cases per 100,000) and the Central division (272.8 per 100,000). The majority of leptospirosis cases were reported between February and May.

### Weekly leptospirosis model

**Fig 2:**
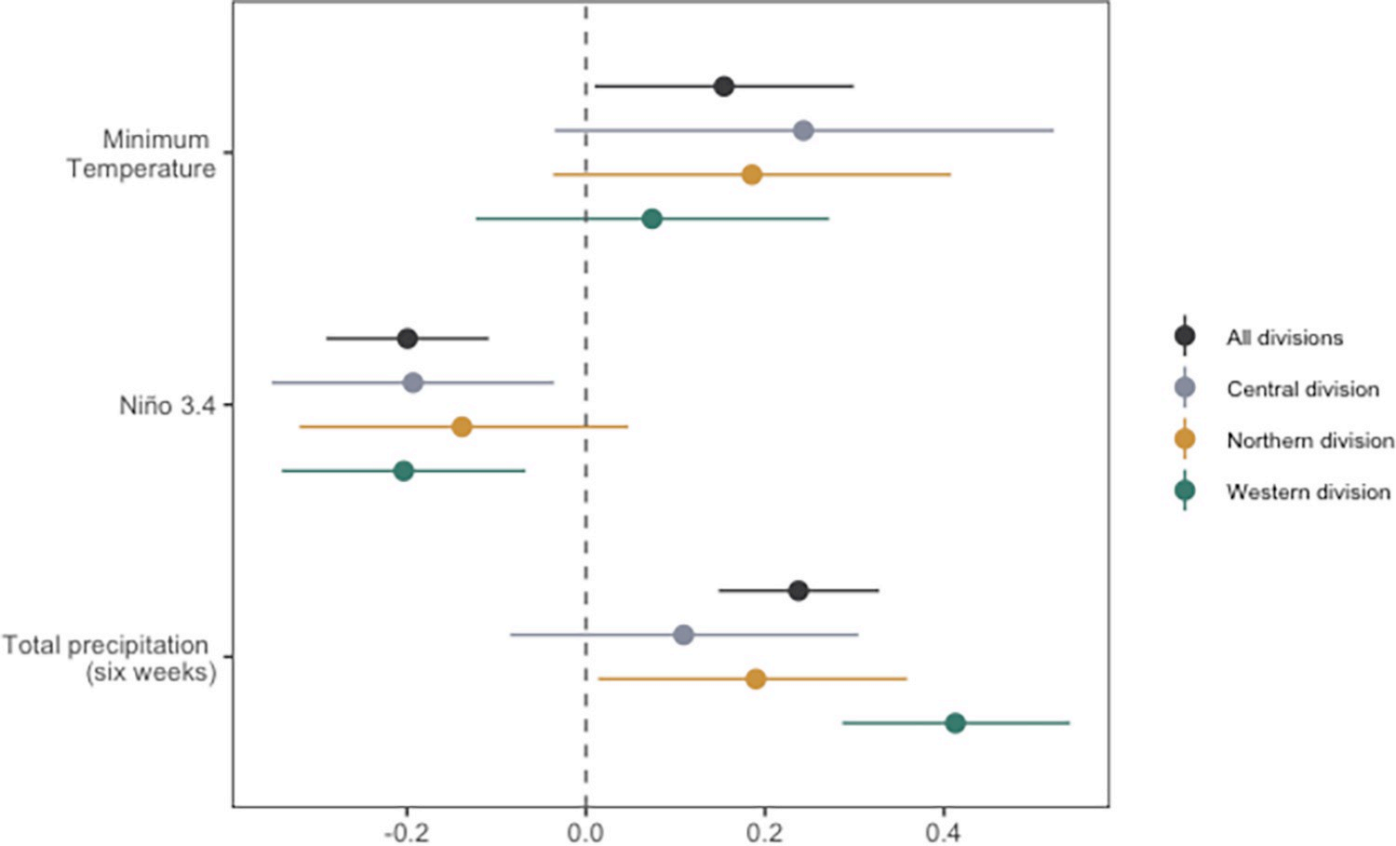
Parameter estimates for explanatory variables for models of ELISA-positive leptospirosis cases per week reported in Fiji from 2007 to 2017 for all divisions (black) and separately by division. Posterior mean and 95% credible intervals are shown for minimum temperature (lagged by one week), total precipitation from the previous six weeks (lagged by one week), and Niño 3.4 (lagged by four weeks).

Using the weekly cases data, a final model was selected (Model 6, Table 2), comprising of weekly random effects and yearly random effects replicated by division (to account for seasonality and unmeasured inter-annual variability by division), minimum temperature lagged by one week (Tmin_t-1_), Niño 3.4 lagged by four weeks (Niño34_t-4_) and total precipitation from the previous six weeks lagged by one week (TotPrcp6_t-1_). Table 2 shows the model goodness of fit results for a series of models with increasing complexity. When minimum temperature was included alone in the model it did not improve model fit (Model 3), however, when included in combination with Niño 3.4 index and total precipitation it did improve model fit (Model 6). Increased levels of precipitation (TotPrcp6_t-1_) was associated with increased leptospirosis risk (0.24 [95% CrI 0.15–0.33]; Fig 2). In addition, we found negative values of the Niño 3.4 index (Niño34_t-4_) to be associated with increased leptospirosis incidence rates (-0.2 [95% CrI -0.29 –-0.11]; Fig 2). Finally, we identified minimum temperature (Tmin_t-1_) to be slightly associated with increases in leptospirosis incidence rates (0.15 [95% CrI 0.01–0.30]; Fig 2).

**Table 2:** Model goodness of fit results for models of ELISA-positive leptospirosis cases per week reported in Fiji from 2007 to 2017. The widely applicable information criterion (WAIC), the cross-validated (CV) mean logarithmic score and the likelihood ratio R_LR_^2^ statistic are shown for models of increasing complexity.

Model		WAIC	CV log score	R_LR_^2^ (%) RE
1	$\alpha +\delta_{sa(t)}+\gamma_{w(t)}$ Baseline model (seasonal and inte-rannual random effects)	5328	1.426	0
2	$\alpha +\delta_{sa(t)}+\gamma_{w(t)}+\beta_{1}x_{st}$ Baseline + Tmin (t-1)	5325	1.425	0.1
3	$\alpha +\delta_{sa(t)}+\gamma_{w(t)}+\beta_{2}x_{st}$ Baseline + Niño34 (t-4)	5302	1.419	1.8
4	$\alpha +\delta_{sa(t)}+\gamma_{w(t)}+\beta_{3}x_{st}$ Baseline + TotPrcp6 (t-1)	5298	1.418	1.4
5	$\alpha +\delta_{sa(t)}+\gamma_{w(t)}+\beta_{2}x_{st}+\beta_{3}x_{st}$ Baseline + Niño34 (t-4) + TotPrcp6 (t-1)	5279	1.413	2.8
6	$\alpha +\delta_{sa(t)}+\gamma_{w(t)}+\beta_{1}x_{st}+\beta_{2}x_{st}+\beta_{3}x_{st}$ Baseline + Tmin (t-1) + Niño34 (t-4) + TotPrcp6 (t-1)	5276	1.412	2.9

Tmin: Minimum temperature (lagged by one week); Niño34: Niño 3.4 (lagged by four weeks); TotPrcp6: Total precipitation from the previous six weeks (lagged by one week).

We explored several different precipitation indicators (e.g. total precipitation and number of heavy rainfall days), and identified total precipitation from the previous six weeks (with a one-week lag) as the best precipitation indicator to explain the variation in leptospirosis cases in Fiji (S1 Table). However, there was only a small improvement compared with other precipitation indicators such as the number of very heavy rainfall days (P20). The mean consecutive wet days was found not to be a good indicator of leptospirosis cases in this model. In addition, SPI and SPEI did not offer any improvements in model fit compared with total precipitation and very heavy rainfall days (S1 Table). We also explored different measures of ENSO and found that Niño 3.4 index was a better predictor than the Niño 4 index. Finally, comparing minimum temperature and maximum temperature we found that minimum temperature showed improved model fit.

**Fig 3:**
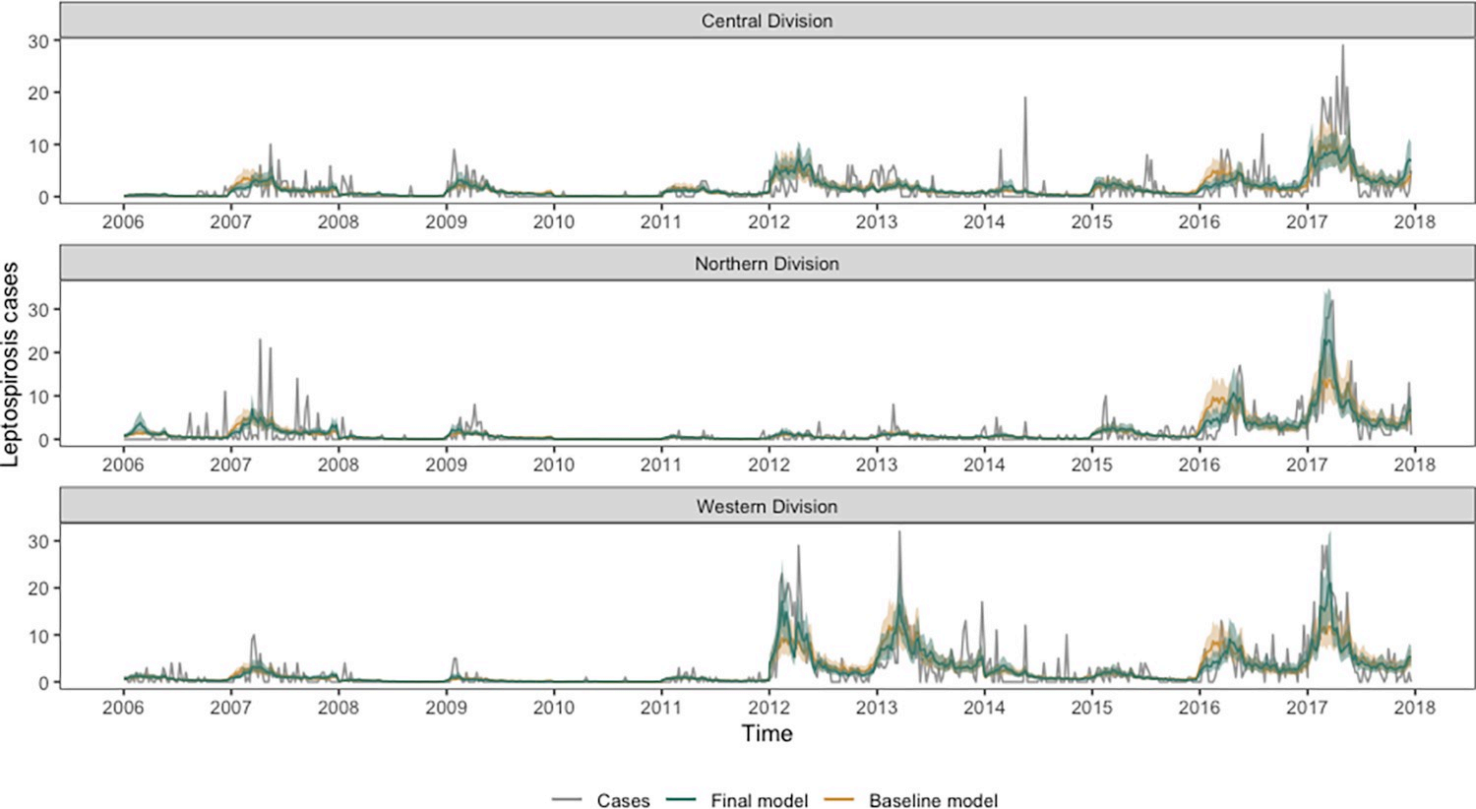
Model posterior estimates for models of ELISA-positive leptospirosis cases per week reported in Fiji from 2007 to 2017 by division. Observed ELISA-positive cases (grey line), posterior model mean (green line) and 95% credible intervals (green shading) are shown for the best performing model which included total precipitation, minimum temperature and Niño 3.4. The random effect only model is shown as an orange dashed line.

The time series of observed cases and model fit is shown in Fig 3. The final model is better able to capture the outbreak peaks (e.g., in 2012 and 2013 in the Western division, in 2016 in the Central, Northern and Western division, and in 2017 in the Western and Northern division) compared with the baseline model (Model 1; which includes only random effects). This is highlighted in Fig C in S1 Text, which shows the relative difference between the baseline and final model. In 58.1% (362 out of 623) of weeks the full model performed better than the random effects model (256 out of 623; 41.1%). However, the model does not appear to capture the peaks in the Central division as well as in the Northern and Western division. The posterior marginal contribution of the seasonal random effects decreased with the inclusion of the climatic indicators (Fig D in S1 Text), demonstrating that climate is accounting for some of the seasonal variation that is observed. However, overall, the posterior marginal contribution of the inter-annual random effects did not appear to shrink towards zero with the inclusion of climatic indicators, indicating that there are other non-climatic factors influencing interannual variability in leptospirosis incidence (Fig E in S1 Text), including, but not limited to, limitations with data quality. Using the parameter estimates associated with the climate variables from the best performing model, we extracted the variation in leptospirosis incidence accounted for by the combined impact of total precipitation, minimum temperature, and the Niño 3.4 index (Fig F in S1 Text). This plot suggests that before 2012, the climatic conditions may have been suitable for a leptospirosis outbreak, particularly in the Western and Northern division in 2008 and 2009, but this is not reflected in the case data. It also shows that in the Central division the role of climate appears to be more consistent compared with the other two divisions.

### Weekly leptospirosis model by division

To understand differences in parameter estimates between the divisions, we explored three separate models for each division. When broken down by division we observed similar trends in the parameter estimates (Fig 2). We found that minimum temperature was still weakly positively associated with leptospirosis cases, but not significant in each division. In addition, we found that negative values of the Niño 3.4 index were still associated with increased cases in all divisions (although not significant in the Northern division). Total precipitation was found to be more strongly associated with leptospirosis cases in the Western division, compared with the Northern and Central divisions, and not statistically significant in the Central division. The R^2^_LR_ for each division model indicates that the climate covariates better account for leptospirosis variation in the Western division compared with the Northern and Central divisions. In the Western division the R^2^_LR_ explained an additional 6.7% of the variation compared with the baseline model, whilst for the Central and Northern division the R^2^_LR_ was 1.6% and -1.2%, respectively (Table B in S1 Text). The negative values indicate that for the Northern division, the baseline model explained more of the variation than the full model.

### Monthly leptospirosis model

As a sensitivity analysis we explored how the model estimates differed by changing temporal scale. We aggregated cases to the monthly scale and performed model selection and evaluation for climate covariates. At the monthly scale, we found that the best performing model included total precipitation from the previous two months (no lag), and Niño 3.4 index (two-month lag; Table C in S1 Text; Fig G in S1 Text). These are similar to the climate covariates identified at the weekly scale. However, minimum temperature was no longer found to increase model fit, and there was no positive association between minimum temperature and leptospirosis cases at the monthly scale. The time series for observed and modelled cases is shown in Fig H in S1 Text. Once again, the final model is better able to capture the outbreak peaks compared to the baseline model (random effects only). However, it does not appear to capture the variability as well in the Central division. The monthly model is better able to capture the variability in the data compared with the weekly data (R^2^_LR_ for the final weekly model was 2.9%, compared with 7.4% for the monthly model; Table B in S1 Text), which may be due to the increased heterogeneity in the data at the weekly scale. Again, looking at the model goodness of fit results, the difference in R^2^_LR_ for each division model shows that climate information in the Western division better explains the variability compared with the Northern and Central division. In the Western division the R^2^_LR_ explained an additional 19.8% of the variation compared with the baseline model, whilst for the Central and Northern division this was 5.8% and -3.0% respectively (Table B in S1 Text). Finally, we also explored how the modelling results changed if we moved from a division level to the whole country. We identified that the same climate covariates at the country level as the division level and saw very little difference in the parameter estimates (Fig G in S1 Text).

## Discussion

Climate is known to influence the timing and size of leptospirosis outbreaks. However, the role of specific climate factors has not been well quantified in Fiji. In the present study, we explored the role of different climatic indicators, including precipitation, temperature, and ENSO, on leptospirosis risk. The results from this study further our understanding of the effect of climatic variables on leptospirosis outbreaks in Fiji, which may allow for a more targeted public health approach in the future.

In this study we found total precipitation in the preceding six weeks, lagged by one week, was the best precipitation indicator, and was positively associated with leptospirosis cases. This supports previous studies that showed precipitation was an important driver of leptospirosis outbreaks, in Fiji and elsewhere [4,19,20,28]. Precipitation, particularly periods of heavy precipitation, is a risk factor for leptospirosis as it can lead to increased environmental exposure to bacteria. We identified six weeks as the best time period, suggesting that cumulative precipitation is important—rather than a few days of sudden heavy rain, and that this cumulative rainfall impacts leptospirosis cases in the following week. In addition to total precipitation, we also found that the number of very heavy rainfall days (number of days in a period where rainfall exceeded 20mm), also over six weeks, was strongly associated with leptospirosis cases, indicating the importance of exploring different climatic indicators in different settings. Minimum temperature, lagged by one week, was also found to be positively associated with leptospirosis risk. Several studies have previously identified that temperature may have a role in leptospirosis outbreaks [47–51], although this appears to be context specific, as other studies have not found this association in different settings [24,52,53]. Laboratory-based studies have identified that increased temperature is associated with increased *Leptospira* survival. However, at very high temperatures (>42°C) survival has been shown to be reduced [21,22], and one study also found some evidence of reduced survival at >30°C [54]. Further studies are required to understand these temperature-dependent relationships as climate change is leading to an increase in frequency of extreme temperatures. The short lag time observed in the present study suggests that temperature may play a role by changing human behaviour and how humans interact with the environment (i.e., in warmer conditions there is more recreational water activity and changes in agricultural activity), rather than increased *Leptospira* survival. In addition, we found that negative values of the Niño 3.4 index (lagged by four weeks) were associated with leptospirosis outbreaks. This suggests that La Niña phases are associated with increased leptospirosis risk in Fiji, and a similar result was observed in New Caledonia [24]. In Fiji, La Niña is associated with increased rainfall and flooding events, which the model suggests may impacts leptospirosis risk the following month. However, given ENSO events occur on average every four years, a longer time series is required to be able to fully understand the relationship between ENSO and leptospirosis risk.

We explored the weekly model separately by division and we found similar trends observed in the parameter estimates, which lends support to the overall weekly model. However, we did find some variation by division, with the model better able to capture cases in the Western division compared with the Northern and Central divisions. Flooding events and tropical cyclones appear to be correlated with increased rainfall in the Western and Northern divisions, however, in the Central division rainfall appeared to be more consistent and less correlated with these events. This suggests that the role of climate on leptospirosis risk may differ by division, or that tropical cyclones and flooding events are not well captured with the precipitation data in the Central division. In the present study, we only had case information by division. By aggregating by division we assume that risk is homogenous across this region, however, these are large areas which encompass many different environmental, and socio-demographic settings. There are likely large differences in the transmission pathways, risk factors and environment between the divisions, therefore, climate may be acting differently in each division, and within each division. For example, risk factors are known to differ in urban, peri-urban, and rural settings, and this was not known for cases [18,29,55]. Additionally, natural environmental factors, such as the proximity of rivers and floodplains, influence the likelihood of experiencing a leptospirosis outbreak in a community. Inadequate sanitation and waste disposal are also risk factors for leptospirosis, and these are linked to poverty. Poverty, particularly in urban areas in Fiji, is known to be associated with a high seroprevalence of leptospirosis [56]. In addition to environmental and socio-economic differences between divisions, the importance of different animal hosts, and therefore transmission pathways and risk factors, has been shown to vary geographically and by ethnic group [29,55]. For example, in urban settings, exposure to livestock was associated with a high risk of infection, which is hypothesised to be a result of closer contact between animals and humans. Furthermore, as many as 19 different serovars and 11 different animal hosts have been identified in Fiji [12,57]. Certain serovars are more commonly associated with certain animal hosts, and serovars also have different pathogenicity associated with them, and disease severity may also vary as a result. Finally, it is known that control practices, for example, rodent control, have taken place in Fiji, although the timing and exact location are not known, and so these are not accounted for in the model. Therefore, due to the complex process driving the transmission of leptospirosis in Fiji, it is likely that the importance and influence of the climatic factors vary depending on geographic and environmental settings. To untangle these differences, enhanced spatial resolution of surveillance data, along with detailed case and serovar information, would be required. Despite this, we were still able to identify climatic drivers likely to be important in explaining leptospirosis variation in Fiji.

As is common for studies of leptospirosis using routine data streams, the results from our analysis are limited by the surveillance data available. In this study, we used surveillance data for leptospirosis over 12 years and only included ELISA-positive cases. This requires individuals to be unwell, report to healthcare, and for samples to be sent for diagnostic testing. It is known that the majority of leptospirosis infections result in mild or asymptomatic infection. In addition, Fiji experiences coinciding outbreaks of leptospirosis and dengue, which share similar clinical manifestations. This is further compounded, as, to conserve testing capability, once a leptospirosis outbreak has been declared, it is recommended that diagnosis is done based on case definitions and clinical judgement [34]. Therefore, the cases and the outbreaks reported by the ELISA-positive data are likely to be a small fraction of the true cases occurring in Fiji [58]. Furthermore, changes in case detection, case reporting and diagnostic capabilities have occurred over time. The number of cases has been increasing in recent years, with several large outbreaks occurring. This may be due to real increases in the number of cases, or due to enhanced surveillance and testing capabilities over time or enhanced clinical suspicion following the release of new leptospirosis guidelines in 2016. Finally, case detection and reporting are likely to vary by division, due to differences in healthcare access, health-seeking behaviours, access to laboratory diagnosis, and clinical and public health capacity. This may have contributed to the differences observed between divisions on the importance of the different climate factors.

We found that the inclusion of climate covariates within the model better captured the outbreak peaks than the random effects only model (e.g., in 2012 and 2013 in the Western division, in 2016 in the Central, Northern and Western division, and in 2017 in the Western and Northern division). We also found that the full model performed better overall than the random effects only model. This suggests that climate information explains some of the seasonal and inter-annual variation in leptospirosis cases and demonstrates the potential use of climate information within an early warning system for leptospirosis. However, looking at the overall yearly random effects, the interannual variation changed very little with the inclusion of climate covariates. This suggests other non-climatic or unknown factors were important in driving interannual variation in cases, and this may also include limitations with the data quality. To help understand both seasonal and interannual drivers of leptospirosis transmission, improved surveillance and spatially explicit case data is needed, along with spatially resolved climatic, environmental and socio-economic variables. While climate may be a significant environmental driver of transmission, the model’s current capacity for prediction is limited. This poses a challenge for the development of any climate-based early warning system for leptospirosis in Fiji and highlights the need for enhanced surveillance. The development of a climate-based early warning system would allow for enhanced knowledge of the timing and the severity of outbreaks which would enable public health responses to mitigate outbreaks.

The results from the present study can be interpreted together with the results from previous studies which have explored socio-demographic and environmental risk factors, to form a more complete view of leptospirosis risk in Fiji. In 2013, a seroprevalence study was conducted in Fiji which identified individual demographic risk factors for leptospirosis [18]. This data was further analysed to understand how these risk factors varied geographically [29,56]. Seroprevalence studies have the advantage that they can capture the prevalence of previous infections, including asymptomatic and mildly symptomatic cases. However, they are a snapshot at one point in time, and are unable to explore how these risk factors vary over time or at different times of the year. Despite the limitations associated with surveillance data, it is longitudinal data which allows for the effect of climate on leptospirosis risk to be explored over time, as was done in the present study. Together, these studies can be thought of as moving towards a precision public health approach, providing more specific insights into differences in incidence between “populations”, “place” and “time”, and a more complete picture of leptospirosis risk in Fiji. Precision public health can be defined as using the best available data to target interventions more efficiently and effectively [59]. This is particularly important in resource-limited settings such as Fiji, as being able to accurately target interventions can provide a cost-effective strategy. In this study, we show how quantifying the effect of climate on weekly surveillance data, combined with the knowledge of those individuals who are most at risk [18], and those “hotspot” locations where prevalence is particularly high [29], can be used to target interventions to those most vulnerable and at risk. These measures could include targeted health promotion and awareness (i.e., encouraging the use of personal protective equipment and covering cuts and abrasions), raising awareness and clinical suspicion, hospital preparedness and ensuring diagnostic capabilities.

Weekly case data is more sensitive to collection processes and reporting delays, and we observed a lot of heterogeneity in the reported weekly cases. Therefore, as a sensitivity analysis, we aggregated the weekly case data into monthly case data and explored how the temporal scale affected our results. We found very similar results, suggesting that there was good agreement between the models. However, minimum temperature was no longer found to be associated with leptospirosis cases at the monthly scale. In the weekly model, we identified that minimum temperature was associated with a one-week lag, suggesting that the effect of temperature occurred on a short time scale, and therefore, it may no longer be observed at a monthly time scale. This suggests that using weekly case data may allow the detection of climatic drivers within short timeframes. However, the choice of temporal scale depends on the research question and the data available. Using weekly case data is more computationally intensive, and many climate covariates are not readily available at the weekly scale. Using a monthly time scale may reduce the heterogeneity in the data as it will be less sensitive to small fluctuations in sample collection and reporting delays. This may allow long-term trends to be more easily identified, and a monthly time scale may be better suited for the development of an early warning system, particularly for neglected zoonotic diseases where surveillance is limited. A summary of the advantages and disadvantages is shown in Table 3.

**Table 3:** Summary of the advantages and disadvantages of fitting a climate-driven statistical model to weekly and monthly surveillance data.

	Advantages	Disadvantages
**Weekly case data**	• Finer temporal resolution, which allows for the effects of climate on disease to be detected at finer time scales, which may be more reflective of disease transmission processes.	• Often not available. • Climatic indicators are often available at monthly scales. • More computationally intensive. • More heterogeneity and uncertainty in the data (“noise”). For diseases and settings where surveillance is very thorough and complete, weekly case data is preferable. However, for diseases such as leptospirosis, where there are varying reporting delays, changes in reporting over time and space, trends may be more apparent at monthly time scales. • Shorter time scales are not always useful, one week does not enable enough time to react and anticipate outbreaks
• **Monthly case data**	• More readily available • Aggregated data may allow for trends to be more apparent and stronger and therefore may be easier to identify long-term trends • More computationally efficient–particularly important if you have high spatial resolution • May be more useful for early warning systems, as the associations may be more robust	• May hide real patterns and trends • Short term effects of climate on infection may not be apparent in the data

In summary, we were able to quantify the association between different climate variables and leptospirosis incidence in Fiji. This study furthers our understanding of how climate affects leptospirosis outbreaks and combined with previous studies exploring the geographical distribution and sociodemographic risk factors, allows us to move towards a precision public health framework. This contributes to our understanding of the climatic risk factors and may allow for more targeted public health interventions in the future. This study also highlights that enhanced surveillance in the future may allow for further studies which untangle the spatial effects of climate on leptospirosis risk, and this is a necessary first step to allow for the development of an early warning system in the future. This will be increasingly important given that climate change in Fiji is predicted to lead to increased rainfall and extreme weather events, and leptospirosis will likely continue to pose a significant burden in this region.

## Supporting information

S1 Table Model goodness of fit results from the climatic indicators and lags included within the study. (XLSX)

S1 Text Fig A in S1 Text. Comparison between leptospirosis cases in Fiji (shown in red) and reported prolonged fever in Fiji from the WHO Pacific Syndromic Surveillance System (shown in blue; [36]) between 2013 and 2017. Table A in S1 Text. Flooding and Tropical Cyclones recorded in Fiji (Central, Western and Northern Division) by the Emergency Events Database (EM-DAT). Fig B in S1 Text. Weekly rainfall from Laucala Bay (Central division), Nadi Airport (Western Division) and Labasa Airfield (Northern Division) between 2006–2017. Red arrows indicate flooding and tropical cyclones recorded in Fiji by the Emergency Events Database (EM-DAT). Fig C in S1 Text. Relative improvement in model fit between the random effect only model and full model. Blue bars represent weeks when the model fit of the full model was better than the random effects only model (i.e., the difference between the observed versus model fitted cases was smaller for the full model compared with the random effects model; n = 362). Red bars represent weeks when the model fit of the random effects only model was better than the full model (n = 256). At zero there is no difference between the two models, and they performed equivalently (n = 5). Fig D in S1 Text. Weekly random effects for the random effect (RE) only model, shown in orange, and the final model which included precipitation, Niño 3.4 and minimum temperature shown in blue. Fig E in S1 Text. Yearly random effects for the random effect (RE) only model (dashed lines) and the final model which included precipitation, Niño 3.4 and minimum temperature (solid lines), for the Central (green), Northern (yellow) and Western (grey) divisions. Fig F in S1 Text. Comparison of (A) the climate variables and model parameter estimates, (B) the full model including seasonal and interannual random effects, and (C) weekly leptospirosis cases reported in Fiji between 2006 and 2017 by division. For (A), the three climate coefficients were extracted from the best performing model and then using the timeseries of total precipitation, minimum temperature and Niño 3.4 indicator, we multiplied the climate coefficients to extract the contribution of the climate covariates to the overall leptospirosis incidence rate estimates. Table B in S1 Text. Likelihood ratio RLR2 statistics are shown for weekly and monthly division models. Table C in S1 Text. Model goodness of fit results for models of ELISA-positive leptospirosis cases per month reported in Fiji from 2007 to 2017. The widely applicable information criterion (WAIC), the cross-validated (CV) mean logarithmic score, and the likelihood ratio RLR2 statistic are shown for models of increasing complexity. Fig G in S1 Text. Parameter estimates for explanatory variables for monthly cases of leptospirosis in Fiji from 2006 to 2017 for the overall country model (black; which included monthly and yearly random effects) and for the division-level model (grey; which included monthly random effects replicated by division, and yearly random effects). Posterior mean and 95% credible intervals are shown for minimum temperature (lagged by one month), total precipitation from the previous two months, and Niño 3.4 lagged by two months. Fig H in S1 Text. Model posterior distributions for monthly leptospirosis cases in Fiji between 2006 and 2017 by division. Observed cases (grey line), posterior mean (green line) and 95% credible intervals (green shading) are shown for the best performing model which included total precipitation and Niño 3.4. The random effect only model is shown as an orange dashed line.
(DOCX)

## References

[1] Costa F, Hagan JE, Calcagno J, Kane M, Torgerson P, Martinez-Silveira MS, et al. Global Morbidity and Mortality of Leptospirosis: A Systematic Review. PLoS Neglected Tropical Diseases. 2015. doi: 10.1371/journal.pntd.0003898 26379143 PMC4574773

[2] Torgerson PR, Hagan JE, Costa F, Calcagno J, Kane M, Martinez-Silveira MS, et al. Global Burden of Leptospirosis: Estimated in Terms of Disability Adjusted Life Years. PLOS Neglected Tropical Diseases. 2015;9: e0004122. doi: 10.1371/journal.pntd.0004122 26431366 PMC4591975

[3] Lau CL. Human Leptospirosis in Oceania. In: Loukas A, editor. Neglected Tropical Diseases—Oceania. Cham: Springer International Publishing; 2016. pp. 177–192. doi: 10.1007/978-3-319-43148-2_7

[4] Lau CL, Smythe LD, Craig SB, Weinstein P. Climate change, flooding, urbanisation and leptospirosis: Fuelling the fire? Transactions of the Royal Society of Tropical Medicine and Hygiene. 2010. doi: 10.1016/j.trstmh.2010.07.002 20813388

[5] Levett PN. Leptospirosis. Clinical Microbiology Reviews. 2001;14: 296–326. doi: 10.1128/CMR.14.2.296-326.2001 11292640 PMC88975

[6] WHO. Human leptospirosis: guidance for diagnosis, surveillance and control. World Health Organisation. 2003;45: 292–292. doi: 10.1590/S0036-46652003000500015

[7] Haake DA, Levett PN. Leptospirosis in humans. Current topics in microbiology and immunology. 2015;387: 65–97. doi: 10.1007/978-3-662-45059-8_5 25388133 PMC4442676

[8] Bharti A, Nally J, Ricaldi J, … MM-TL, undefined. Leptospirosis: a zoonotic disease of global importance. Elsevier. 2003. Available: http://www.sciencedirect.com/science/article/pii/S1473309903008302. 10.1016/s1473-3099(03)00830-2 14652202

[9] Lau CL, DePasquale JM. Leptospirosis Diagnostic Challenges, American Samoa. Emerg Infect Dis. 2012;18: 2079–2081. doi: 10.3201/eid1812.120429 23171761 PMC3557872

[10] Picardeau M, Bertherat E, Jancloes M, Skouloudis AN, Durski K, Hartskeerl RA. Rapid tests for diagnosis of leptospirosis: Current tools and emerging technologies. Diagnostic Microbiology and Infectious Disease. 2014;78: 1–8. doi: 10.1016/j.diagmicrobio.2013.09.012 24207075

[11] Musso D, La Scola B. Laboratory diagnosis of leptospirosis: A challenge. Journal of Microbiology, Immunology and Infection. 2013;46: 245–252. doi: 10.1016/j.jmii.2013.03.001 23639380

[12] Guernier V, Goarant C, Benschop J, Lau CL. A systematic review of human and animal leptospirosis in the Pacific Islands reveals pathogen and reservoir diversity. 2018;12. doi: 10.1371/journal.pntd.0006503 29758037 PMC5967813

[13] Ellis WA. Animal Leptospirosis. In: Adler B, editor. Leptospira and Leptospirosis. Berlin, Heidelberg: Springer Berlin Heidelberg; 2015. pp. 99–137. doi: 10.1007/978-3-662-45059-8_6

[14] Harrison NA, Fitzgerald WR. Leptospirosis—can it be a sexually transmitted disease? Postgraduate Medical Journal. 1988;64: 163–164. doi: 10.1136/pgmj.64.748.163 3174532 PMC2428802

[15] Andre-Fontaine G, Aviat F, Thorin C. Waterborne Leptospirosis: Survival and Preservation of the Virulence of Pathogenic Leptospira spp. in Fresh Water. Current Microbiology. 2015;71: 136–142. doi: 10.1007/s00284-015-0836-4 26003629

[16] Casanovas-Massana A, Pedra GG, Wunder EA, Diggle PJ, Begon M, Ko AI. Quantification of Leptospira interrogans Survival in Soil and Water Microcosms. Appl Environ Microbiol. 2018;84. doi: 10.1128/AEM.00507-18 29703737 PMC6007094

[17] Trueba G, Zapata Mena S, Madrid K, Cullen P, Haake D. Cell aggregation: A mechanism of pathogenic Leptospira to survive in fresh water. International microbiology: the official journal of the Spanish Society for Microbiology. 2004;7: 35–40. 15179605

[18] Lau CL, Watson CH, Lowry JH, David MC, Craig SB, Wynwood SJ, et al. Human Leptospirosis Infection in Fiji: An Eco-epidemiological Approach to Identifying Risk Factors and Environmental Drivers for Transmission. Picardeau M, editor. PLOS Neglected Tropical Diseases. 2016;10: e0004405. doi: 10.1371/journal.pntd.0004405 26820752 PMC4731082

[19] Mwachui MA, Crump L, Hartskeerl R, Zinsstag J, Hattendorf J. Environmental and Behavioural Determinants of Leptospirosis Transmission: A Systematic Review. PLoS Neglected Tropical Diseases. 2015;9. doi: 10.1371/journal.pntd.0003843 26379035 PMC4574979

[20] Naing C, Reid SA, Aye SN, Htet NH, Ambu S. Risk factors for human leptospirosis following flooding: A meta-analysis of observational studies. PLOS ONE. 2019;14: e0217643. doi: 10.1371/journal.pone.0217643 31141558 PMC6541304

[21] Parker J, Walker M. Survival of a pathogenic Leptospira serovar in response to combined in vitro pH and temperature stresses. Veterinary Microbiology. 2011;152: 146–150. doi: 10.1016/j.vetmic.2011.04.028 21592682

[22] Noguchi H. Further study on the cultural conditions of leptospira (spirochaeta) icterohaemorrhagiae. J Exp Med. 1918;27: 593–608. doi: 10.1084/jem.27.5.593 19868228 PMC2125879

[23] Fiji Meteorological Service. Fiji Climate Change and Health Adaptation: Fiji’s current climate variability, climate change and future projections. Feb 2015 [cited 18 May 2022]. Available: https://www.health.gov.fj/wp-content/uploads/2018/03/Fiji-Climate-Change-and-Health-Adaption-Symposium-Booklet.pdf.

[24] Weinberger D, Baroux N, Grangeon JP, Ko AI, Goarant C. El Niño Southern Oscillation and Leptospirosis Outbreaks in New Caledonia. PLoS Neglected Tropical Diseases. 2014;8: 1–7. doi: 10.1371/journal.pntd.0002798 24743322 PMC3990495

[25] Chand SS, Walsh KJE. Influence of ENSO on Tropical Cyclone Intensity in the Fiji Region. Journal of Climate. 2011;24: 4096–4108. doi: 10.1175/2011JCLI4178.1

[26] Red Cross Red Crescent Climate Centre. Climate change impacts on health and livelihoods: Fiji assessment. Apr 2021 [cited 18 May 2022]. Available: https://www.climatecentre.org/wp-content/uploads/RCRC_IFRC-Country-assessments-FIJI.pdf.

[27] Lotto Batista M, Rees EM, Gómez A, López S, Castell S, Kucharski AJ, et al. Towards a leptospirosis early warning system in northeastern Argentina. Journal of The Royal Society Interface. 2023;20: 20230069. doi: 10.1098/rsif.2023.0069 37194269 PMC10189304

[28] Togami E, Kama M, Goarant C, Craig SB, Lau C, Ritter JM, et al. A Large Leptospirosis Outbreak following Successive Severe Floods in Fiji, 2012. The American journal of tropical medicine and hygiene. 2018. doi: 10.4269/ajtmh.18-0335 30141390 PMC6159581

[29] Mayfield HJ, Lowry JH, Watson CH, Kama M, Nilles EJ, Lau CL. Use of geographically weighted logistic regression to quantify spatial variation in the environmental and sociodemographic drivers of leptospirosis in Fiji: a modelling study. The Lancet Planetary Health. 2018;2: e223–e232. doi: 10.1016/S2542-5196(18)30066-4 29709286 PMC5924768

[30] Nations United. List of Small Island Developing States (SIDS): Sustainable Development Knowledge Platform. 1 Jan 2019 [cited 6 Jan 2022]. Available: https://sustainabledevelopment.un.org/topics/sids/list.

[31] International Monetary Fund. Republic of Fiji: 2021 Article IV Consultation-Press Release; Staff Report; and Statement by the Executive Director for the Republic of Fiji. In: IMF [Internet]. 1 Dec 2021 [cited 2 Jun 2023]. Available: https://www.imf.org/en/Publications/CR/Issues/2021/12/03/Republic-of-Fiji-2021-Article-IV-Consultation-Press-Release-Staff-Report-and-Statement-by-510770.

[32] The Fijian Government. Fiji Bureau of Statistics Releases 2017 Census Results. In: The Fijian Government [Internet]. 10 Jan 2018 [cited 4 Jan 2022]. Available: https://fiji.gov.fj.

[33] Ministry of Foreign Affairs. Republic of Fiji: second national communication to the United Nations Framework Convention on Climate Change. 2014 [cited 2 Aug 2019]. Available: https://unfccc.int/resource/docs/natc/fjinc2.pdf.

[34] Ministry of Health and Medical Services, Republic of Fiji. Communicable Disease Surveillance and Outbreak Response Guidelines. 2016.

[35] MOHMS. Clinical Guidelines for Diagnosis and Management of Leptospirosis. Republic of Fiji Ministry of Health and Medical Services. 2016.

[36] World Health Organisation. Pacific syndromic surveillance reports. 2019 [cited 13 Jun 2023]. Available: https://www.who.int/westernpacific/emergencies/surveillance/pacific-islands-surveillance.

[37] National Oceanic and Atmospheric Administration. Climate Prediction Center—Monitoring & Data: Current Monthly Atmospheric and Sea Surface Temperatures Index Values. 2022 [cited 18 May 2022]. Available: https://www.cpc.ncep.noaa.gov/data/indices/.

[38] World Meteorological Organization, Expert Team on Climate Change Detection and Indices. Guidelines on Analysis of extremes in a changing climate in support of informed decisions for adaptation. 2009 [cited 18 May 2022]. Available: https://community.wmo.int/climate-change-detection-and-indices.

[39] Romanello M, McGushin A, Napoli CD, Drummond P, Hughes N, Jamart L, et al. The 2021 report of the Lancet Countdown on health and climate change: code red for a healthy future. The Lancet. 2021;398: 1619–1662. doi: 10.1016/S0140-6736(21)01787-6 34687662 PMC7616807

[40] Beguería S, Vicente-Serrano SM, Reig F, Latorre B. Standardized precipitation evapotranspiration index (SPEI) revisited: parameter fitting, evapotranspiration models, tools, datasets and drought monitoring. International Journal of Climatology. 2014;34: 3001–3023. doi: 10.1002/joc.3887

[41] Beguería S, Vicente-Serrano SM. SPEI: Calculation of the Standardised Precipitation-Evapotranspiration Index. 2017.

[42] EM-DAT. The international disasters database. 2022 [cited 11 Jul 2022]. Available: https://www.emdat.be/.

[43] Gelman A, Hwang J, Vehtari A. Understanding predictive information criteria for Bayesian models. Stat Comput. 2014;24: 997–1016. doi: 10.1007/s11222-013-9416-2

[44] Gneiting T, Raftery AE. Strictly Proper Scoring Rules, Prediction, and Estimation. Journal of the American Statistical Association. 2007;102: 359–378. doi: 10.1198/016214506000001437

[45] Wei T, Simko V, Levy M, Xie Y, Jin Y, Zemla J, et al. corrplot: Visualization of a Correlation Matrix. 2021. Available: https://CRAN.R-project.org/package=corrplot.

[46] Fox J, Weisberg S, Price B, Adler D, Bates D, Baud-Bovy G, et al. car: Companion to Applied Regression. 2022. Available: https://CRAN.R-project.org/package=car.

[47] Desvars A, Jégo S, Chiroleu F, Bourhy P, Cardinale E, Michault A. Seasonality of Human Leptospirosis in Reunion Island (Indian Ocean) and Its Association with Meteorological Data. Ojcius DM, editor. PLoS ONE. 2011;6: e20377. doi: 10.1371/journal.pone.0020377 21655257 PMC3105052

[48] Chadsuthi S, Modchang C, Lenbury Y, Iamsirithaworn S, Triampo W. Modeling seasonal leptospirosis transmission and its association with rainfall and temperature in Thailand using time-series and ARIMAX analyses. Asian Pacific Journal of Tropical Medicine. 2012;5: 539–546. doi: 10.1016/S1995-7645(12)60095-9 22647816

[49] Joshi YP, Kim E-H, Cheong H-K. The influence of climatic factors on the development of hemorrhagic fever with renal syndrome and leptospirosis during the peak season in Korea: an ecologic study. BMC Infectious Diseases. 2017;17. doi: 10.1186/s12879-017-2506-6 28592316 PMC5463320

[50] Silva AEP, Latorre M do RD de O, Chiaravalloti Neto F, Conceição GM de S. Temporal trends in leptospirosis incidence and association with climatic and environmental factors in the state of Santa Catarina, Brazil. Ciênc saúde coletiva. 2022;27: 849–860. doi: 10.1590/1413-81232022273.45982020 35293463

[51] Ehelepola NDB, Ariyaratne K, Dissanayake WP. The correlation between local weather and leptospirosis incidence in Kandy district, Sri Lanka from 2006 to 2015. Glob Health Action. 2019;12: 1553283. doi: 10.1080/16549716.2018.1553283 31154987 PMC6327921

[52] Coelho MSZS, Massad E. The impact of climate on Leptospirosis in São Paulo, Brazil. Int J Biometeorol. 2012;56: 233–241. doi: 10.1007/s00484-011-0419-4 21369729

[53] Deshmukh P, Narang R, Jain J, Jain M, Pote K, Narang P, et al. Leptospirosis in Wardha District, Central India—Analysis of hospital based surveillance data. Clinical Epidemiology and Global Health. 2019;7: 102–106. doi: 10.1016/j.cegh.2018.02.005

[54] Chang SL, Buckingham M, Taylor MP. Studies on Leptospira Icterohaemorrhagiae: IV. Survival in water and sewage: destruction in water by halogen compounds, synthetic detergents, and heat. The Journal of Infectious Diseases. 1948; 256–266. 10.1093/infdis/82.3.256 18864110

[55] Lau CL, Mayfield HJ, Lowry JH, Watson CH, Kama M, Nilles EJ, et al. Unravelling infectious disease eco-epidemiology using Bayesian networks and scenario analysis: A case study of leptospirosis in Fiji. Environmental Modelling & Software. 2017;97: 271–286. doi: 10.1016/j.envsoft.2017.08.004

[56] Mayfield HJ, Smith CS, Lowry JH, Watson CH, Baker MG, Kama M, et al. Predictive risk mapping of an environmentally-driven infectious disease using spatial Bayesian networks: A case study of leptospirosis in Fiji. Carvalho MS, editor. PLOS Neglected Tropical Diseases. 2018;12: e0006857. doi: 10.1371/journal.pntd.0006857 30307936 PMC6198991

[57] Togami E. Animal & Human Leptospirosis In A High Transmission Setting In Fiji. Public Health Theses, Yale University. 2016; 28.

[58] Rees EM, Lau CL, Kama M, Reid S, Lowe R, Kucharski AJ. Estimating the duration of antibody positivity and likely time of Leptospira infection using data from a cross-sectional serological study in Fiji. PLOS Neglected Tropical Diseases. 2022;16: e0010506. doi: 10.1371/journal.pntd.0010506 35696427 PMC9232128

[59] Horton R. Offline: In defence of precision public health. The Lancet. 2018;392: 1504. doi: 10.1016/S0140-6736(18)32741-7 30496048

