# Supplementary material for: Quantifying the relationship between climatic indicators and leptospirosis incidence in Fiji: A modelling study
Source: PLOS Glob Public Health. 2023 Oct 11;3(10):e0002400. doi: 10.1371/journal.pgph.0002400 (PMC10566718; doi:10.1371/journal.pgph.0002400)
Supplement: S1 Text — Fig A in S1 Text. Comparison between leptospirosis cases in Fiji (shown in red) and reported prolonged fever in Fiji from the WHO Pacific Syndromic Surveillance System (shown in blue; [36]) between 2013 and 2017. Table A in S1 Text. Flooding and Tropical Cyclones recorded in Fiji (Central, Western and Northern Division) by the Emergency Events Database (EM-DAT). Fig B in S1 Text. Weekly rainfall from Laucala Bay (Central division), Nadi Airport (Western Division) and Labasa Airfield (Northern Division) between 2006–2017. Red arrows indicate flooding and tropical cyclones recorded in Fiji by the Emergency Events Database (EM-DAT). Fig C in S1 Text. Relative improvement in model fit between the random effect only model and full model. Blue bars represent weeks when the model fit of the full model was better than the random effects only model (i.e., the difference between the observed versus model fitted cases was smaller for the full model compared with the random effects model; n = 362). Red bars represent weeks when the model fit of the random effects only model was better than the full model (n = 256). At zero there is no difference between the two models, and they performed equivalently (n = 5). Fig D in S1 Text. Weekly random effects for the random effect (RE) only model, shown in orange, and the final model which included precipitation, Niño 3.4 and minimum temperature shown in blue. Fig E in S1 Text. Yearly random effects for the random effect (RE) only model (dashed lines) and the final model which included precipitation, Niño 3.4 and minimum temperature (solid lines), for the Central (green), Northern (yellow) and Western (grey) divisions. Fig F in S1 Text. Comparison of (A) the climate variables and model parameter estimates, (B) the full model including seasonal and interannual random effects, and (C) weekly leptospirosis cases reported in Fiji between 2006 and 2017 by division. For (A), the three climate coefficients were extracted from the best performing [file pgph.0002400.s002.docx]

# Supporting information

######
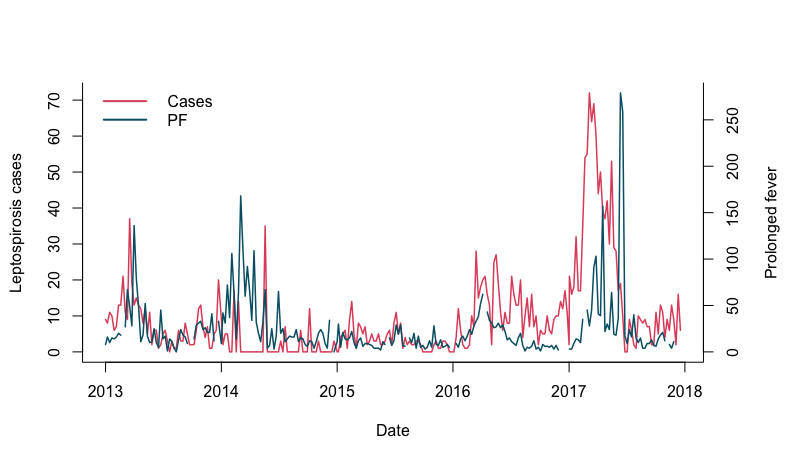
 Fig. A. Comparison between leptospirosis cases in Fiji (shown in red) and reported prolonged fever in Fiji from the WHO Pacific Syndromic Surveillance System [shown in blue; (36)] between 2013 and 2017.

######

######

######

######

######

######

######

######

######

######

######

###### Table A. Flooding and Tropical Cyclones recorded in Fiji (Central, Western and Northern Division) by the Emergency Events Database (EM-DAT).

| Year | Date | Disaster Type | Division |
| --- | --- | --- | --- |
|  |  |  |  |
| 2007 | 3/02/2007 - 20/02/2007 | Riverine flood (origin heavy rain) | Central, Northern, Western |
| 2007 | 9/03/2007 - 12/03/2007 | Flash flood | Central, Northern, Western |
| 2008 | 28/01/2008 - 29/01/2008 | Cyclone "Gene" (associated disaster flooding) | Central, Northern, Western |
| 2009 | 8/01/2009 - 19/01/2009 | Riverine flood (origin heavy rain) | Central, Northern, Western |
| 2009 | 14/12/2009 - 15/12/2009 | Cyclone "Mick" (associated disaster flooding) | Central, Western |
| 2010 | 14/03/2010 - 16/03/2010 | Cyclone "Tomas" (associated disaster flooding) | Central, Northern, Western |
| 2012 | 22/01/2012 - 06/02/2012 | Riverine flood (origin heavy rain & tropical depression) | Western |
| 2012 | 29/03/2012 - 30/03/2012 | Riverine flood (origin heavy rain & tropical depression) | Western |
| 2012 | 16/12/2012 - 18/12/2012 | Cyclone "Evan" | Northern, Western |
| 2016 | 20/02/2016 - 21/02/2016 | Cyclone "Winston" (associated disaster flooding) | Central, Northern, Western |
| 2016 | 04/04/2016 - 07/04/2016 | Cyclone "Zena" (associated disaster flooding) | Central, Western |

######

######
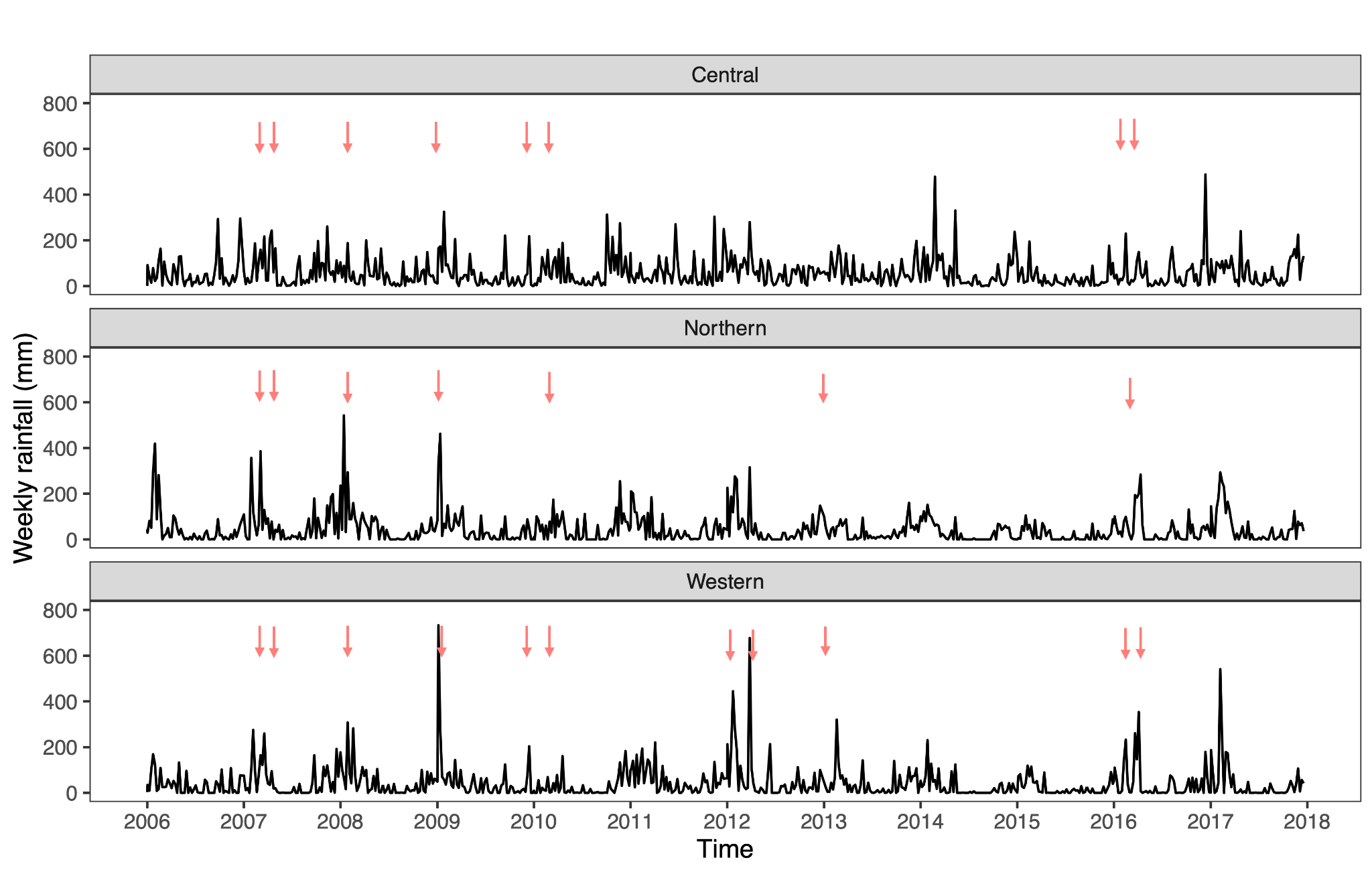
 Fig. B. Weekly rainfall from Laucala Bay (Central division), Nadi Airport (Western Division) and Labasa Airfield (Northern Division) between 2006-2017. Red arrows indicate flooding and tropical cyclones recorded in Fiji by the Emergency Events Database (EM-DAT).

######

######
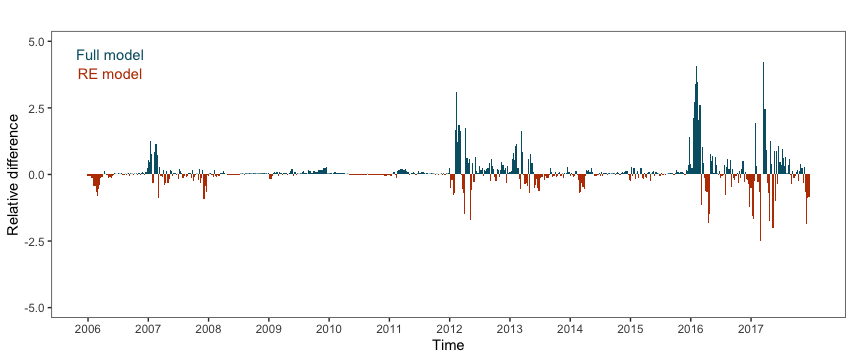


###### Fig. C. Relative improvement in model fit between the random effect only model and full model. Blue bars represent weeks when the model fit of the full model was better than the random effects only model (i.e., the difference between the observed versus model fitted cases was smaller for the full model compared with the random effects model; n=362). Red bars represent weeks when the model fit of the random effects only model was better than the full model (n=256). At zero there is no difference between the two models, and they performed equivalently (n=5).


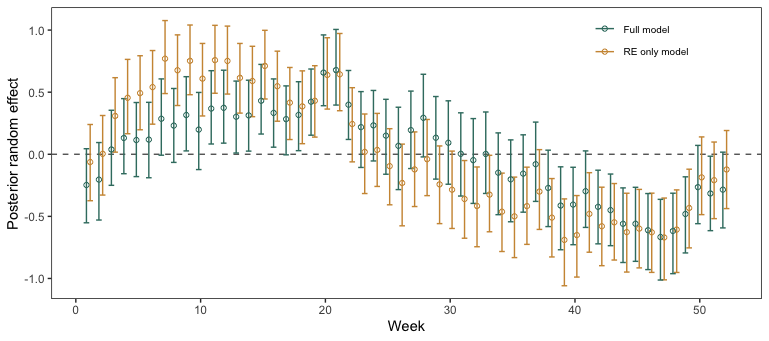


###### Fig. D. Weekly random effects for the random effect (RE) only model, shown in orange, and the final model which included precipitation, Niño 3.4 and minimum temperature shown in blue.


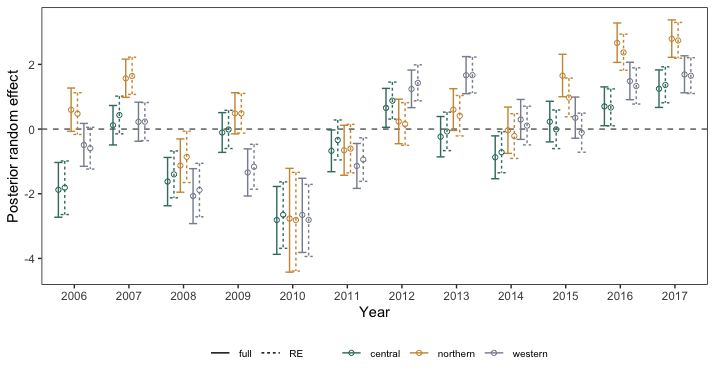


###### Fig. E. Yearly random effects for the random effect (RE) only model (dashed lines) and the final model which included precipitation, Niño 3.4 and minimum temperature (solid lines), for the Central (green), Northern (yellow) and Western (grey) divisions.

######
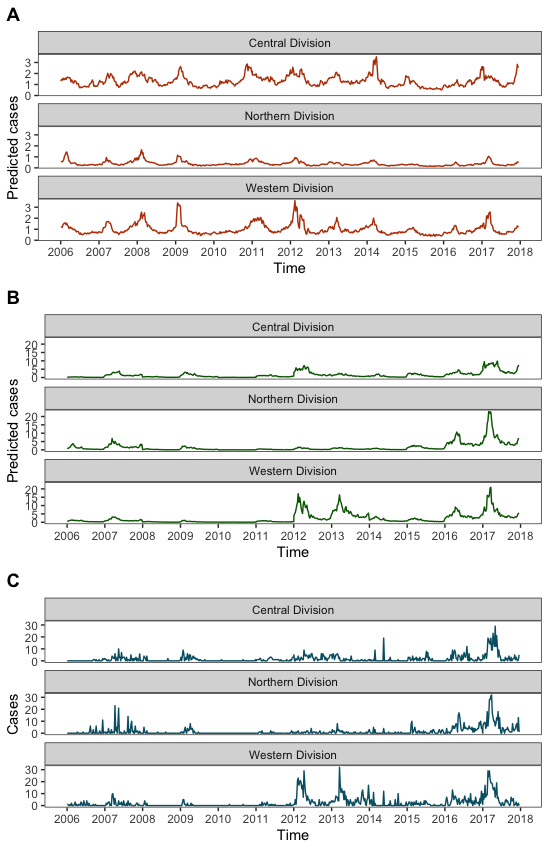


###### Fig. F. Comparison of (A) the climate variables and model parameter estimates, (B) the full model including seasonal and interannual random effects, and (C) weekly leptospirosis cases reported in Fiji between 2006 and 2017 by division. For (A), the three climate coefficients were extracted from the best performing model and then using the timeseries of total precipitation, minimum temperature and Niño 3.4 indicator, we multiplied the climate coefficients to extract the contribution of the climate covariates to the overall leptospirosis incidence rate estimates.

######

######

######

###### Table B. Likelihood ratio R_LR_^2^ statistics are shown for weekly and monthly division models.

| **Model** | **R_LR_^2^ (%) (weekly) Null** | **R_LR_^2^ (%) (weekly) RE** | **R_LR_^2^(%) (monthly) Null** | **R_LR_^2^(%) (monthly) RE** |
| --- | --- | --- | --- | --- |
| All divisions | 47.4 | 2.9 | 68.0 | 7.4 |
| Central division | 39.8 | 1.6 | 58.1 | 5.8 |
| Western division | 54.9 | 6.7 | 77.8 | 19.8 |
| Northern division | 40.3 | -1.2 | 60.9 | -3.0 |

######

###### Table C. Model goodness of fit results for models of ELISA-positive leptospirosis cases per month reported in Fiji from 2007 to 2017. The widely applicable information criterion (WAIC), the cross-validated (CV) mean logarithmic score, and the likelihood ratio R_LR_^2^ statistic are shown for models of increasing complexity.

| **Model** | | **WAIC** | **CV log score** | **R_LR_^2^ RE(%)** |
| --- | --- | --- | --- | --- |
| 1 | $\alpha+ \delta_{t,s}+ \gamma_{w}$  Baseline model (seasonal and inter-annual random effects) | 2191 | 2.541 | 0 |
| 2 | $\alpha+ \delta_{t,s}+ \gamma_{w} + x_{1tsw}$  Baseline + Tmin (t-1) | 2192 | 2.543 | -0.2 |
| 3 | $\alpha+ \delta_{t,s}+ \gamma_{w} + x_{2tsw}$  Baseline + Niño34 (t-2) | 2177 | 2.525 | 3.7 |
| 4 | $\alpha+ \delta_{t,s}+ \gamma_{w} + x_{3tsw}$  Baseline + TotPrcp2 | 2168 | 2.514 | 4.7 |
| 5 | $\alpha+ \delta_{t,s}+ \gamma_{w} + x_{2tsw}+ x_{3tsw}$  Baseline + Niño34 (t-2) + TotPrcp2 | 2159 | 2.504 | 7.4 |
| 6 | $\alpha+ \delta_{t,s}+ \gamma_{w} + x_{1tsw}+ x_{2tsw}+ x_{3tsw}$  Baseline + Tmin (t-1) + Niño34 (t-2) + TotPrcp2 | 2160 | 2.505 | 7.4 |

######

######

######
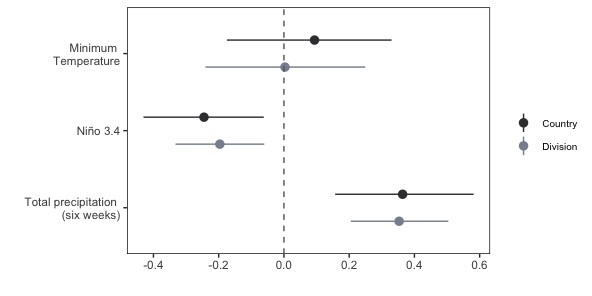


###### Fig. G. Parameter estimates for explanatory variables for monthly cases of leptospirosis in Fiji from 2006 to 2017 for the overall country model (black; which included monthly and yearly random effects) and for the division-level model (grey; which included monthly random effects replicated by division, and yearly random effects). Posterior mean and 95% credible intervals are shown for minimum temperature (lagged by one month), total precipitation from the previous two months, and Niño 3.4 lagged by two months.


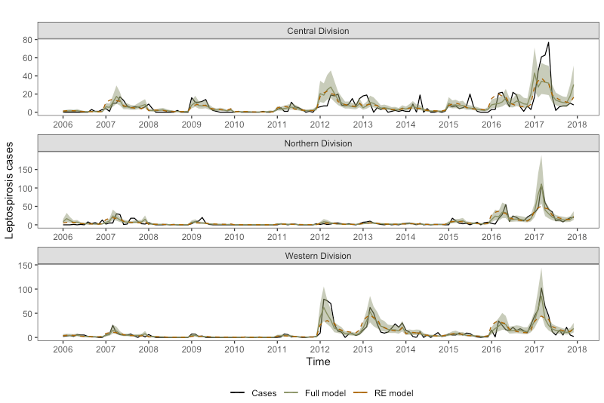


###### Fig. H. Model posterior distributions for monthly leptospirosis cases in Fiji between 2006 and 2017 by division. Observed cases (grey line), posterior mean (green line) and 95% credible intervals (green shading) are shown for the best performing model which included total precipitation and Niño 3.4. The random effect only model is shown as an orange dashed line.

# 
